# False positives and false negatives measure less than 0.001% in labeling ssDNA with osmium tetroxide 2,2’-bipyridine

**DOI:** 10.3762/bjnano.7.135

**Published:** 2016-10-12

**Authors:** Anastassia Kanavarioti

**Affiliations:** 1 Yenos Analytical LLC, El Dorado Hills, CA, USA

**Keywords:** α-hemolysin, capillary electrophoresis, DNA sequencing, high-performance liquid chromatography, nanopores, osmium tetroxide bipyridine, osmylated oligos, osmylation, ssDNA

## Abstract

Osmium tetroxide 2,2’-bipyridine (OsBp) is known to react with pyrimidines in ssDNA and preferentially label deoxythymine (T) over deoxycytosine (C). The product, osmylated DNA, was proposed as a surrogate for nanopore-based DNA sequencing due to OsBp’s “perfect” label attributes. Osmylated deoxyoligos translocate unassisted and measurably slow via sub-2 nm SiN solid-state nanopores, as well as via the alpha-hemolysin (α-HL) pore. Both nanopores discriminate clearly between osmylated and intact nucleobase; α-HL was also shown to discriminate between osmylated T and osmylated C. Experiments presented here confirm that the kinetics of osmylation are comparable for short oligos and long ssDNA and show that pyrimidine osmylation is practically complete in two hours at room temperature with less than 15 mM OsBp. Under the proposed labeling conditions: deoxyoligo backbone degradation measures less than 1/1,000,000; false positives such as osmylated deoxyadenine (A) and osmylated deoxyguanine (G) measure less than 1/100,000; false negatives, i.e., unosmylated C measure less than 1/10,000; and unosmylated T must measure substantially lower than 1/10,000 due to the 27-fold higher reactivity of T compared to C. However, osmylated C undergoes degradation that amounts to about 1–2% for the duration of the labeling protocol. This degradation may be further characterized, possibly suppressed, and the properties of the degradation products via nanopore translocation can be evaluated to assure base calling quality in a DNA sequencing effort.

## Introduction

At the time the human genome was completed in 2001 [1–2], it was becoming clear that the relationships between genes, noncoding regions, epigenetic modifications, and the transcriptome were intertwined and led to health vs disease in complex patterns. A large effort in academia, government, and industry pushed DNA sequencing to next-generation technologies [3] that flourished exponentially due to technological advancements in parallelization, miniaturization, and bioinformatics. DNA sequencing was and still is an extension of the Sanger sequencing-by-synthesis (SBS) method [4]. Nevertheless, SBS limitations [5], and the need for single-molecule sequencing at a fast speed, low cost, and accurate base-calling has led to the exploration of nanopore-based solutions and other platforms [6–7].

Physical scientists embraced nanopore technology for a plethora of applications [8–10]. The advantage of protein pores is that they are well defined and highly reproducible, whereas the advantage of solid-state nanopores is that they can be manufactured to desirable dimensions. Protein pores such as α-HL, a modified version of the *Mycobacterium smegmatis* porin A (MspA), and the Ph29 connector channel have been investigated as single-molecule sensing devices for ssDNA, RNA, dsDNA, and proteins [11–13]. The concept of nanopore-based sequencing, patented in 1998 [14], is based on applying a potential across an open pore embedded within an insulating membrane that separates two compartments filled with electrolyte. Influenced by the electric field, the electrolyte ions traverse the pore producing a constant current. Also led by the applied field, a nucleic acid in one compartment moves through the pore to the other compartment and obstructs the current in a sequence information-rich manner [15–17].

Nanopore-based sequencing of ssDNA relies on current modulation attributed to the single-file passing of the nucleotide bases A, G, C, or T through the pore. Typically, nanopores interact with a short sequence, and therefore the observed current obstruction may not be attributed to a single base [11,18]. From a chemical point of view, the bases are not that different from each other and yield comparable current levels, as shown by experiments where the ssDNA was immobilized within the pore [19–21]. Discrimination is further reduced when the nucleic acid traverses the pore in speeds that correspond microseconds per base − speeds that are too fast for detection by current instrumentation [11]. To remedy the situation, work mostly with α-HL and MspA has yielded engineered versions thereof with improved DNA sensing capabilities [12,22–23], as well as the incorporation of Ph29 polymerase, or other appropriate enzymes at the lip of the nanopores. This acts to slow down translocation by binding to the strand and moves it one base at a time through the pore [24–25].

**Scheme 1:**
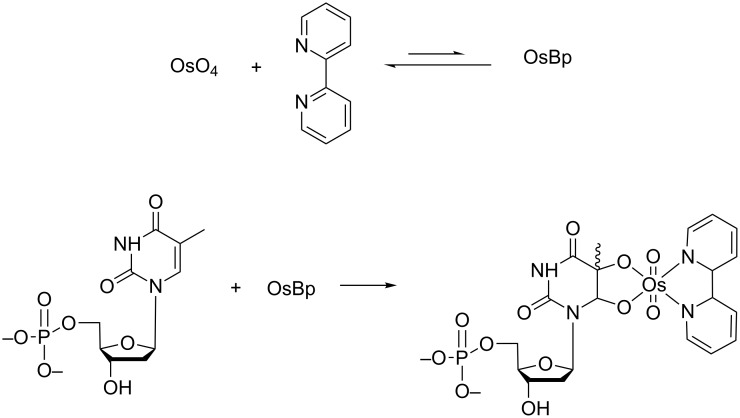
A 1:1 mixture of osmium tetroxide with 2,2’-bipyridine in solution forms a reactive complex (OsBp), which in a second step reacts with the C5–C6 double bond of a pyrimidine (thymidine monophosphate (dTMP) shown here) to form the osmylated-pyrimidine. Two topoisomers, in about a ratio 2:1, are produced by addition from the top or the bottom of the pyrimidine ring. It is only the final product that carries the strong absorbance in the range of 300 to 320 nm, and hence discriminates itself from the other components by UV–vis. Even though OsBp is a minor component [30], for simplicity, the OsBp is referred to the 1:1 labeling mixture. Hence, the OsBp concentration (listed under reaction conditions) refers to the initial concentration of each of the two components. In this context, “osmylation” is referred to as the conjugation reaction of OsBp with a nucleobase.

To resolve the issues of similar chemical structure and unreadable speed of translocation [26], we considered a different approach, namely selective labeling of ssDNA. Historically, selective labeling was evaluated in the 1960s in an effort to image DNA conjugated with contrast agents by electron microscopy in order to elucidate sequence from the images [27–29]. This was tested within this work and it was found that one of the contrast agents, a 1:1 mixture of osmium tetroxide and 2,2’-bipyridine, exhibited extraordinary properties, and was considered to be the “perfect” label (Scheme 1). These attributes are (i) room temperature reactivity at mM concentration, (ii) no loss of label upon standing, (iii) superior selectivity for T over C by a factor of 27, (iv) undetectable degradation of the DNA strand under the labeling conditions, and (v) undetectable reactivity towards the purines (false positives). Most importantly OsBp reactivity towards T or C was found to be independent of composition, sequence, length, and secondary structure of the DNA strand, as shown in [30–32] and also the results herein. For example, oligothymidylates, such as T8, T15 and T20, exhibit fully labeled product formation that is identical to the rate of labeling of an oligoadenylate with one T or, even to osmylation of the monomer, the deoxythymidylic acid triphosphate, 5’dTTP [30]. The across-the-board equal reactivity leads to predictable osmylation of any DNA without prior sequence knowledge. Conjugation of OsBp to a pyrimidine leads to a new chromophore that absorbs in the range of 300 to 320 nm, a range where DNA does not absorb. This last feature enabled the development of a quality control UV–vis assay to confirm the extent of labeling [30–32].

It was hypothesized that a nanopore of suitable size would differentiate between osmylated and bare base due to their major differences in size and structure. Indeed it was recently experimentally confirmed that both 1.6 nm wide SiN nanopores [33] and α-HL [34] allow translocation of an osmylated oligo at strikingly slow, measurable speeds. Current recordings from experiments using 20 nucleotide long deoxyoligos with sequence A_10_XA_9_, where X = A, C, T, 5-MeC, U (deoxyuridine), solidly support the conclusion that α-HL discriminates between osmylated and intact base, as well as among the different osmylated pyrimidines tested. This last observation yields a tentative nanopore-based methodology whereby the osmylated target strand may provide sequencing information on T and C, and the osmylated complementary strand may provide information on A and G.

Undoubtedly a labeled nucleic acid, used as a surrogate for sequencing via electron microscopy or nanopore technology, is required to meet stringent qualifications. These qualifications should ascertain a “perfect” match between intact and osmylated, generally speaking “labeled” nucleic acid, so that sequencing of the labeled nucleic acid can accurately reproduce the sequence of the target polymer. These properties, defined elsewhere [26], include: (i) high solubility in water/electrolyte, (ii) less than 1/10,000 in false positives, (iii) less than 1/10,000 in false negatives, and (iv) negligible degradation of the DNA backbone. In addition, any side reaction that occurs during labeling should be identified and quantified. Experiments are described here to show that under the proposed osmylation conditions, these qualifications are met.

## Results and Discussion

**Figure 1:**
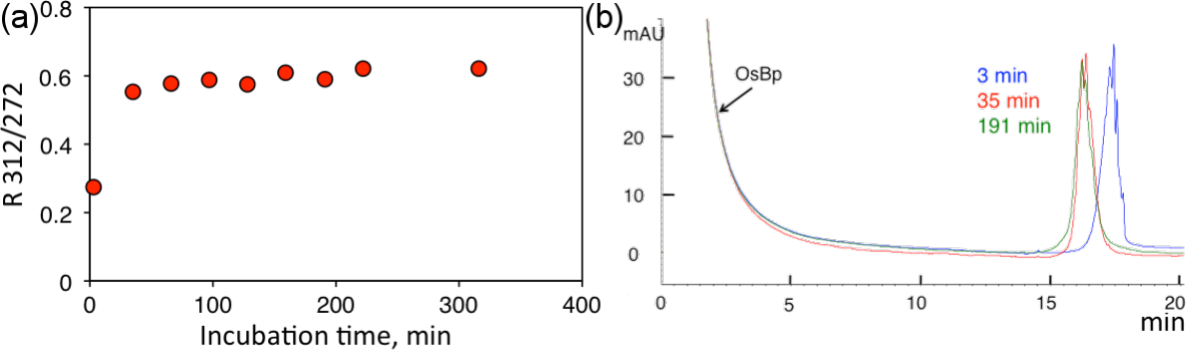
(a) Reactivity profile of a 32-mer deoxyoligo, pGEX3’-A_9_, in aqueous 12.6 mM OsBp at 25 °C monitored automatically by HPLC (see oligo sequence in Table 1). Practically complete osmylation is observed after 40 min. It is noticeable that the peak area of the oligo does not show major changes at 260 nm (shown), or at 272 nm. However, the absorbance at 312 nm starts at 0 and increases with extent of osmylation; *R* minimizes instrument sampling errors. (b) HPLC profiles at 260 nm for selected analyses. The OsBp peak is very large but elutes early and does not interfere with the oligo peak. Because this oligo has 9 pyrimidines, osmylation results in multiple isomers that elute closely together as a broad peak. Nevertheless, the more extensive the osmylation, the earlier the retention time (rt) [30]; this is why there is a rt difference between the profiles at 3 min and at 35 min incubation, but insignificant difference in rt between 35 min and 191 min. Oligo is abbreviated as pGEX3’-A_9_, as the nonadenylated part of this oligo corresponds to the sequence of the PCR primer pGEX 3’.

Earlier studies identified a chromophore, resulting from the formation of the pyrimidine/OsBp bond, which absorbs in the range of 300 to 320 nm where DNA has practically no absorbance [30–32]. These studies also selected two wavelengths and their absorbance ratio (*R* = *A*(λ = 312 nm)/*A*(λ = 272 nm), i.e., the absorbance at 312 nm divided by the absorbance at 272 nm) as optimal for monitoring the osmylation of either T or T + C. Osmylation can be monitored at 312 nm directly, but determining *R* cancels sampling errors. *R* is a direct measure of the extent of osmylation and can be obtained spectrophotometrically after purification/removal of the excess label. In the presence of OsBp, the values of *R* are directly obtained from the DNA peak when the reaction mixture is analyzed and monitored by high performance liquid chromatography (HPLC) or capillary electrophoresis (CE). It turns out that both analytical tools clearly resolve DNA from the OsBp label, which appears as a single peak with the methods used in this report. For the more typical cases where the oligo/DNA has 5 or more pyrimidines, the resolution between intact and osmylated nucleic acid is lost, and the substrate/product appears as a single broad peak. Figure 1 illustrates *R* for the reaction of a 32 nucleotide long oligo with 12.6 mM OsBp in water at 25 °C, as monitored by HPLC. Upon HPLC analysis, OsBp elutes very early, and the osmylated product elutes late, allowing the visualization of complete osmylation (plateau), as well as of any side reactions (additional peaks) that could be attributed to degradation products. Under these conditions, the reaction is quite fast and all pyrimidines have reacted after about 40 min, as evidenced by the plateau. Moreover, no new peaks accumulate over time, as shown by stability studies for up to two weeks at room temperature.

**Table 1:** List of oligos/DNA tested in this study with abbreviations and sequences; all bases deoxy including deoxyuridine (U). *R*1 and *R*2 represent the ratio *R* value upon using protocol A or B, respectively (see Experimental).

Abbreviation	*R* = *A*(λ = 312 nm)/*A*(λ = 272 nm) observed	Sequence

A_20_	0	AAA AAA AAA AAA AAA AAA AA
A_10_GA_9_	0	AAA AAA AAA AGA AAA AAA AA
A_10_TA_9_	*R*1 = *R*2 = 0.11	AAA AAA AAA ATA AAA AAA AA
A_10_CA_9_	*R*2 = 0.11	AAA AAA AAA ACA AAA AAA AA
A_10_C(Me)A_9_	*R*2 = 0.11	AAA AAA AAA AC(Me)A AAA AAA AA
A_10_UA_9_	*R*2 = 0.11	AAA AAA AAA AUA AAA AAA AA
A_10_CA_4_	*R*2 = 0.14	AAA AAA AAA ACA AAA
A_10_UA_4_	*R*2 = 0.14	AAA AAA AAA AUA AAA
16S RNA for pCR primer	*R*1 = 0.64, *R*2 = 1.05	AGA GTT TGA TCC TGG CTC AG
pGEX 3’-A_9_	*R*1 = 0.26, *R*2 = 0.59	CCG GGA GCT GCA TGT GTC AGA GGA AAA AAA AA
M13mp18	*R*1 = 0.75, *R*2 = 1.18	[35]

### Comparable reactivity of oligos and ssDNA

Earlier studies unexpectedly showed the kinetic independence of the labeling process on sequence, composition and length among a large number of tested deoxyoligos with length 8-mer to 99-mer, including the monomers. Note that only fully labeled product formation is kinetically independent of the above parameters, but not the disappearance of the intact oligo, as its kinetics are statistically defined by the number of pyrimidines in the molecule [30]. The absorbance of the osmylated product varies proportionally with the fraction of the osmylated pyrimidines over the total number of bases (*N*_tot_), and the corresponding equation *R* = 2.01·[T(OsBp)+C(OsBp)]/*N*_tot_ was obtained using an oligo training set [31]. Osmylation kinetics of M13mp18, a 7249 nucleotide long circular ssDNA, concluded that this DNA osmylates just like any oligo and led to the conjecture that other ssDNAs may follow suit.

**Figure 2:**
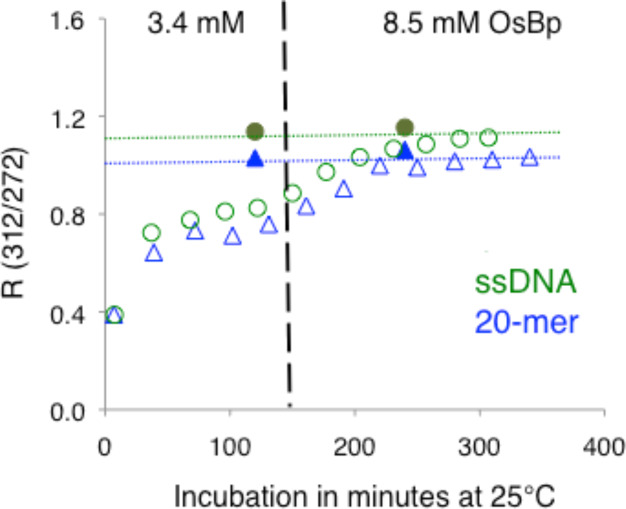
Direct comparison of the kinetics of a 20 nucleotide long deoxyoligo (triangles, PCR primer “16S RNA for”, for simplicity “20-mer”, sequence given in Table 1) and the circular 7249 nucleotide long ssDNA M13mp18 (circles, for simplicity “ssDNA”, see Table 1) as obtained by CE (see Experimental). The data show kinetically comparable reactivity of these two dramatically different materials under three different osmylation conditions, i.e. in 3.4 mM, or in 8.5 mM, or in 13.6 mM OsBp (solid symbols). Dotted lines indicate “theoretical” *R*2 for 100% osmylation, as estimated from equation *R* = 2.01 × (C + T)_tot_/*N*_tot_, obtained earlier using a training set of oligos/DNA [31]. Theoretical *R*2 values at 1.01 and 1.09 and observed *R*2 values at 1.05 and 1.18, respectively for “16S RNA for” and ssM13mp18. *R*2 values are *R*(312/272) upon protocol B osmylation that yields practically 100% T + C labeling (see Experimental).

Figure 2 illustrates the reactivity of M13mp18, and directly compares it with that of a 20 nucleotide long deoxyoligo, the PCR Primer “16S RNA for” (see Table 1). This oligo was selected with T and C composition similar to M13mp18, so that *R* has a comparable value for both. The experiment was conducted by reacting each material with the same concentration of OsBp, initially 3.4 mM to practically osmylate Ts. After 2 h, OsBp was added so that the final concentration was 8.5 mM – a concentration that is suitable for labeling Cs relatively slowly so that the process can be monitored by HPLC or CE. Reactions were also conducted with 13.6 mM OsBp and the reaction mixtures were purified ahead of analysis. It is striking that under the above three different osmylation conditions, the kinetic behavior (see ascending curves, and plateaus) of the oligo and the long DNA are practically identical, suggesting that the same protocol may be used predictably for any ssDNA. The fact that all the data points lie somewhat lower for the oligo compared to M13mp18 is due to the somewhat lower fraction of T and C in the oligo. The ratio T/*N*_tot_ = 0.30 for the 20-mer and 0.33 for M13mp18; (T + C)/*N*_tot_ equals 0.50 for the 20-mer and 0.54 for M13mp18 [31].

### Upper limits for purine osmylation and DNA backbone degradation (false positives)

**Figure 3:**
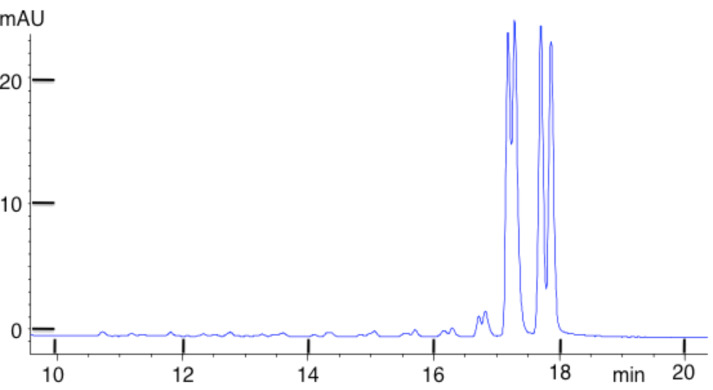
HPLC analysis of deoxyoligos A_10_XA_9_ with X = A, G, C, T. An equimolar mixture of four 20-mers differing only by one base analyzed by IE HPLC on DNAPac. An equimolar mixture of the four oligos appears as a single peak by CE, but as four separate peaks by HPLC using a NaCl gradient in a 25 mM Tris HCl pH 7.0 buffer on a 2 × 250 mm DNAPac PA200 from Dionex.

Capillary zone electrophoresis (CZE) methods were extensively used to study the osmylation kinetics with nucleic acids [30–31]. The characteristic of CZE is that the reagent, OsBp, migrates early with a migration time (mt) that corresponds to neutral molecules, whereas the nucleic acid migrates late and the osmylated nucleic acid migrates in between, when resolution between substrate and product is feasible. More extensive osmylation leads to earlier mt values. The advantage of CZE is that typically nucleic acids share comparable mt irrespective of length and composition, which leads to the convenience of using a single method for all tested materials. The disadvantage is that CZE may not show tentative degradation of the backbone or plausible side reactions. Therefore, ion exchange (IE) HPLC with DNA Pac column from Dionex (see Experimental) was exploited. To our knowledge, this is one of the best columns to resolve oligos primarily based on length and also based on composition. Figure 3 illustrates the last point, namely the resolution of four 20 nucleotide long deoxyoligos that differ only in the 11th nucleotide: A_10_XA_9_ with X = A, T, G, and C, a separation not achieved by CZE.

**Figure 4:**
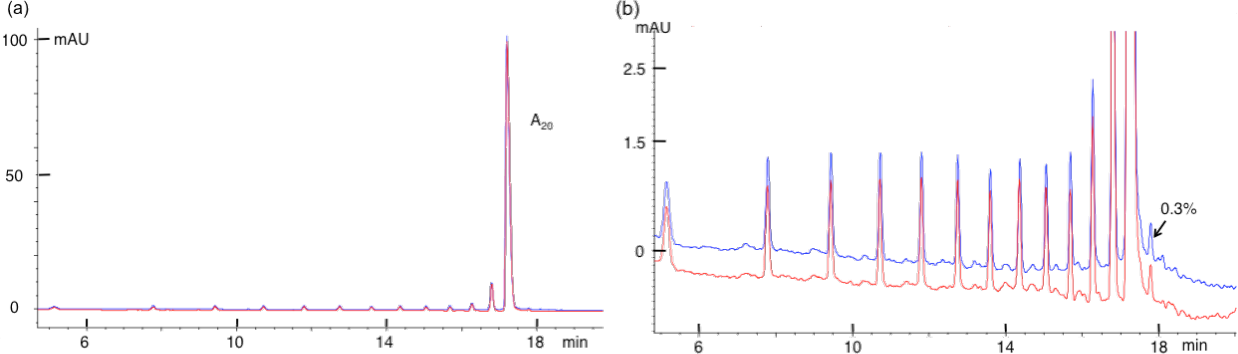
Overlapping HPLC profiles of A_20_ before (red) and after (blue) osmylation to show no detectable reactivity of A with OsBp. (a) Oligo, about 85% pure, in full scale, and (b) the magnification to show the presence of small impurities, the shorter oligos with *n*−1. The chromatography resolves oligos based on length with *n*−1 eluting ahead of *n*. The impurity at 0.3% of the main peak is marked to visualize the detectability level of this specific experiment.

**Figure 5:**
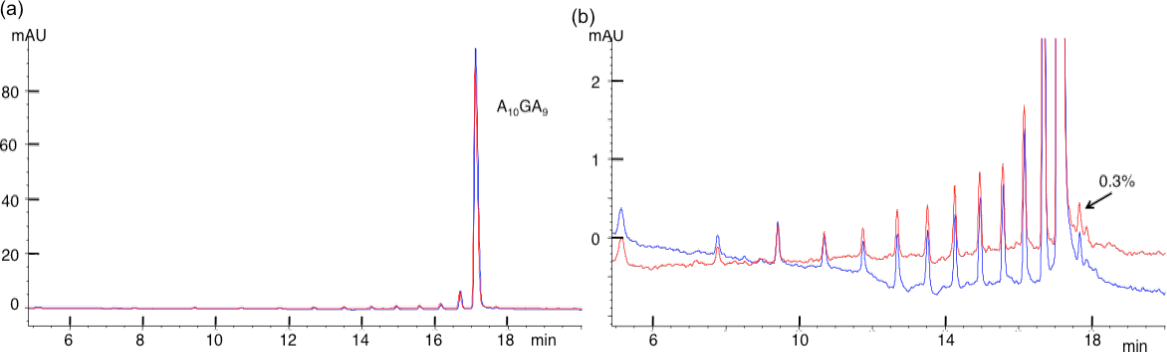
Overlapping HPLC profiles of A_10_GA_9_ before (red) and after (blue) osmylation to show no detectable reactivity of A and G with OsBp. (b) Oligo, about 85% pure, in full scale, and (b) the magnification shows the presence of small impurities, the shorter oligos with *n*−1. The chromatography resolves oligos based on length with *n*−1 eluting ahead of *n*. Please note that all shown oligos of decreasing length from *n*−1 to *n*−10 must contain one G. The impurity at 0.3% of the main peak is marked to visualize the detectability level of this specific experiment.

Figure 4a illustrates the HPLC profile of A_20_ as the major peak preceded by a number of impurities of decreasing relative abundance. These impurities are magnified 40-fold in Figure 4b; they are the oligos with *n* < 20, where the lower the *n*, the earlier the rt. In Figure 4, the red trace represents the intact A_20_ and blue trace represents A_20_ after 12 h osmylation in 14.2 mM OsBp at room temperature. The two traces are superimposable, suggesting that A_20_ does not react and does not degrade under these conditions. Since the small peak after the main component measures 0.3%, one may conclude that a 0.2% peak would also be detectable and estimate that any reaction/degradation would be detectable at or above 0.2%. A comparable behavior is shown by A_10_GA_9_ in Figure 5a,b. Here again osmylation of this oligo does not produce any changes and/or any peaks that would have been detected at levels at or above 0.2%, as per the argument made above for A_20_. Since incubation is 6-times longer than protocol B requires and A_20_ has 20As, then the observation of 0.2% product would have corresponded to a 0.002/20/6 = 1.7/100,000 upper limit for A osmylation and 0.002/6 = 3.3/10,000 = 0.033% for G osmylation in A_10_GA_9_.

A second major conclusion from Figure 4 and Figure 5 is that oligos of *n*−1 length are clearly resolved with this method, which will be exploited later to show that an oligo composed of all four bases exhibits no detectable degradation to shorter oligos during osmylation by protocol B. The conclusion for undetectable osmylation of G is in contrast to observations published elsewhere [36], and it is attributed to the different conditions used in the two studies.

**Figure 6:**
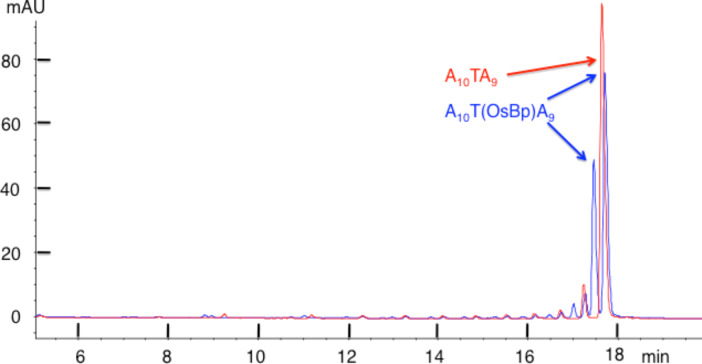
Overlapping IE HPLC profiles of intact and osmylated oligo A_10_TA_9_ to show that the main peak (red) upon osmylation yields two product peaks (blue). This observation is consistent with the notion that OsBp adds from either side of the C5–C6 double bond in T.

**Figure 7:**
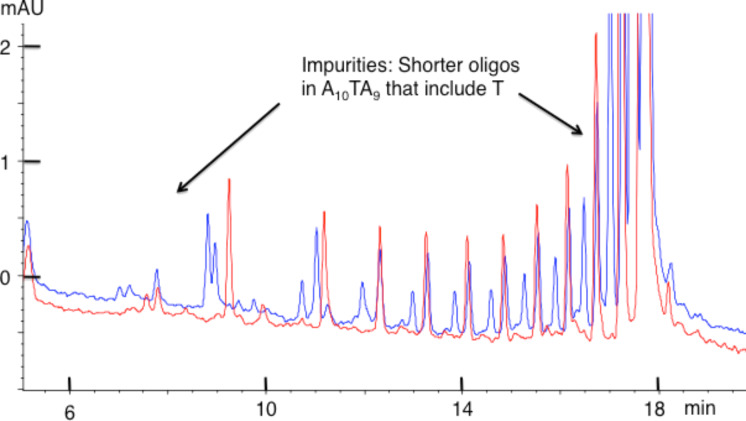
Magnification of Figure 6 to show the impurities present in A_10_TA_9_ and the fact that each oligo (red) that contains a T forms two isomeric product peaks (blue) in an approximate 2:1 ratio. Note that by HPLC the major isomer elutes later than the minor isomer, whereas by CE the major isomer migrates ahead of the minor isomer.

In contrast to the nonreactivity observed with A_20_ and A_10_GA_9_ (see Figure 4 and Figure 5), osmylated A_10_TA_9_ exhibits a completely different HPLC profile compared to the intact oligo (Figure 6). Figure 6 illustrates that the intact 20-mer (red trace) is replaced by two peaks (blue trace) with an approximate 2:1 ratio upon osmylation. Figure 7 (magnification of Figure 6) illustrates that the first major impurity (red) is also replaced by two peaks (blue) and this pattern continues up to about *n* = 11; namely, every impurity peak that includes a T reacts with OsBp and forms two isomeric products in an approximate 2:1 ratio. The formation of two isomers for T(OsBp) was observed by CE and reported earlier [30–31]. Specifically, two isomers are formed by addition of OsBp to the double bond from the top or the bottom of the pyrimidine ring. The HPLC analysis of the osmylated A_10_CA_9_ (not shown) is comparable to Figure 6 and Figure 7.

**Figure 8:**
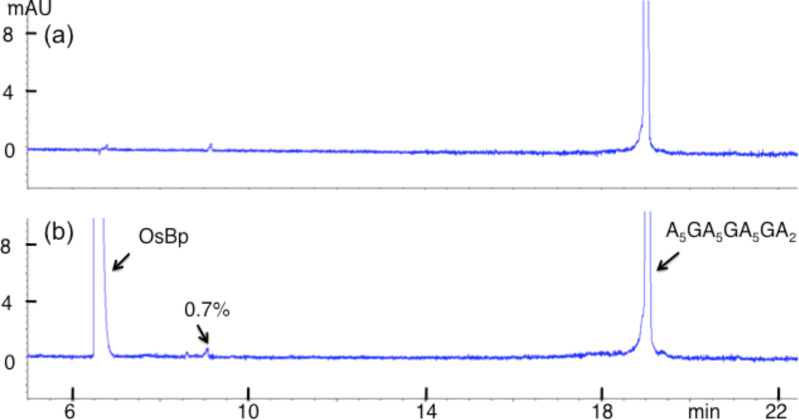
CE profiles of A_5_GA_5_GA_5_GA_2_, a 20-mer with 3 Gs, before and after extensive osmylation to show no detectable reactivity of A and G with OsBp. (a) indicates that this oligo is over 99% pure (b) that the reaction mixture marks the presence of an impurity at 0.7% to show that a reaction product of that level would have been detectable. An osmylation product would have migrated between the oligo and the OsBp peaks (long capillary used here, see Experimental). (a) shows the analysis of the parent oligo at 10 μM concentration and (b) the analysis of the treated oligo at 20 μM concentration.

The above experiment with oligo A_10_GA_9_ (Figure 5) indicated that false positive and backbone degradation on a G base would have been detectable at levels at or above 0.033%. In order to assess a tentative G reactivity at higher sensitivity, we monitored the osmylation of an oligo (A_5_GA_5_GA_5_GA_2_) with 3 Gs by CE. The reaction was attempted in the presence of 12.6 mM OsBp and instead of 2 h, it was monitored for three days (Figure 8). Assuming a limit of 0.2% in product detectability, and accounting for 3 Gs and a 36-fold longer incubation, the upper limit for G reactivity with OsBp under protocol B conditions is 0.002/3/36 = 0.002% or 2/100,000. From this experiment, the limit for A-osmylation is at or less than 0.002/17/36 = 0.3/100,000, in agreement with the earlier experiment (Figure 4). These calculations of 0.3/100,000 for A reactivity and 2/100,000 for G reactivity are upper limits of detectability, and not actual levels of reactivity. This is due to limitations in instrumentation and the specifics of the experiment. These limits are also consistent with undetectable backbone degradation of the over 70 oligos that we have osmylated so far. It is likely that false positives for purine reaction and backbone degradation of purines under the harshest of our osmylation conditions (2 h with <15 mM OsBp in water at room temperature) are below 1/100,000.

**Figure 9:**
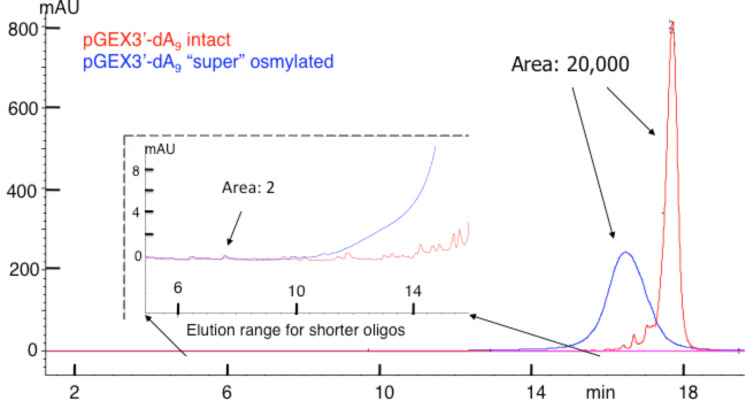
Overlapping HPLC profiles of intact oligo and the corresponding product resulting by “super” osmylation analyzed using a high-load injection. Oligo pGEX3’-dA_9_, a 32-mer (Table 1), is >95% pure, and does not exhibit impurities, i.e., shorter oligos, in the range of 10 to 20-mers (see insert). The osmylated product elutes as a broad peak, but does not exhibit any degradation products, as seen in the insert. The area of a peak with 2 (HPLC units) is marked to visualize the detectability level for a tentative backbone degradation. Note the 32-mer would have eluted at a later time compared to the 20-mers if the amounts and loads were comparable. However, the approximately 50-fold higher oligo load of this experiment results in earlier elution, but this does not alter the elution time and/or resolution of any shorter trace oligos.

To assess reactivity of OsBp towards the backbone of an oligo composed of all four nucleobases, including osmylated pyrimidines, we experimented with an oligo that happened to be of exceptional purity. This oligo is a 32-mer, 3’-end-adenylated PCR primer pGEX3’, abbreviated as pGEX3’A_9_ (Table 1). Figure 9 shows the IE HPLC profile of this 32-mer with the main peak eluting close to 18 min and several impurities eluting between the main peak and rt = 16 min. Since this method is the identical method we used for the analysis of the 20-mers shown in Figures 3–6, any short product oligo with *n* > 10 is expected to elute in the range of 5 min < rt < 16 min. To push the detectability limit, analysis was done on a large sample of intact oligo with an area of 20,468 HPLC units (red trace in Figure 9). A second aliquot from this oligo batch was “super”-osmylated (3 times longer than protocol B) and analyzed immediately after a sample of the intact. The osmylated oligo, as expected, elutes ahead of the intact as a broader peak, due to multiple topoisomers. Even though the tail of the osmylated peak extends towards the 15 min range, any peak produced as the result of backbone breakdown would have been detectable in the range of 5 min < rt < 15 min. Figure 9 shows a magnification of this range and assigns HPLC area to 2 units, attributable to a small visible peak that is present in both materials. This experiment suggests that degradation of the backbone of a 32 nucleotide long oligo that incorporates osmylated pyrimidines and 31 plausible positions for backbone cleavage would be visible at the level of 2/20,135/3/31 = 1/1,000,000, and suggests that OsBp reactivity towards a DNA backbone, osmylated or not, is lower than 1/1,000,000. As previously discussed, this limit is the result of our current instrumentation and other experiments may push this limit even lower.

### Instability of C(OsBp)

There is a literature precedent that suggests that osmylated C deamidates and hydrolyzes to osmylated U [37]. In this work, ion channel recordings for pyrimidine(OsBp) were obtained within a sequence of oligodeoxyadenylates (A_10_XA_9_, where X is deoxypyrimidine), using α-HL nanopores. The response of the nanopore to the presence of the osmylated pyrimidine is dramatically different compared to an intact base. Still C(OsBp) and U(OsBp) exhibit comparable relative current obstruction (*I*_r_/*I*_o_), but different dwell times (τ) at 120 mV in pH 7 buffered 1M KCl [34]. Specifically, *I*_r_/*I*_o_ = 0.12 for both, and corrected per unit mean translocation time τ = 312.5 and τ = 422.5 μs, for C(OsBp) and U(OsBp), respectively [34,38]. In contrast, *I*_r_/*I*_o_ = 0.08 and τ = 102.5 μs for T(OsBp) was obtained under the same conditions. In the context of nanopore-based osmylated ssDNA sequencing, the conversion of C(OsBp) to U(OsBp) may be inconsequential, because these two exhibit, relatively speaking, comparable properties, while both are clearly distinguishable from T(OsBp). Hence a mix-up between U(OsBp) and T(OsBp) may be as unlikely as a mix-up between C(OsBp) and T(OsBp). Still the conversion is undesirable in the context of nanopore-based sequencing of osmylated RNA, where riboC and riboU are the most abundant pyrimidines, especially if the ion-channel recordings of the osmylated riboC and riboU mimic the trends observed with their deoxy counterparts (see above).

To test the tentative transformations of C(OsBp) to U(OsBp), and of C(Me)(OsBp) to T(OsBp), as well as any other instability during the labeling process, we set up osmylation experiments in 13.6 mM OsBp using A_10_CA_9_, A_10_C(Me)A_9_, A_10_CA_4_ and evaluated the stability of the osmylated product over time using both IE HPLC and CE with the long 50 μm × 72 cm capillary (see the Experimental section). In addition, A_10_C(Me)(OsBp)A_9_, A_10_T(OsBp)A_9_ and A_10_U(OsBp)A_9_ were prepared and their stability was tested over time as well (see Supporting Information File 1 Figures S1, S2, and S3, respectively). These materials were also used for identification of tentative byproducts from the presumed hydrolysis of C(OsBp) to U(OsBp) and C(Me)(OsBp) to T(OsBp). We confirmed that C(Me)OsBp, T(OsBp), and U(OsBp) are stable over a couple of weeks at room temperature, whereas C(OsBp) both within the 20-mer and the 15-mer degrades over time. It was also established that the degradation of C(OsBp) is in the range of about 1–2% during the protocol B osmylation and that this degradation does not involve the loss of OsBp.

**Figure 10:**
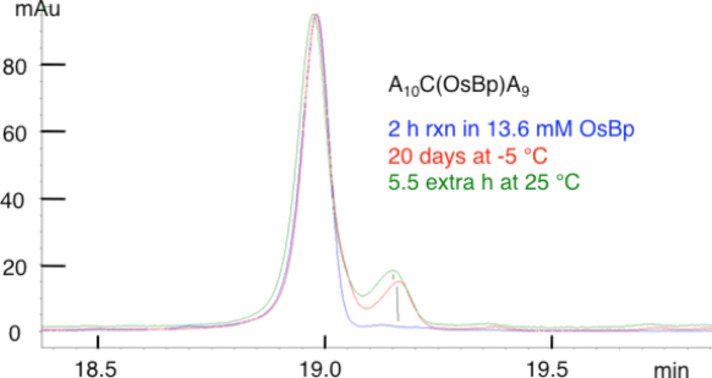
Overlapping CE profiles from stability studies of A_10_C(OsBp)A_9_ in water: Blue, after 2 h reaction in 13.6 mM OsBp and purification by TrimGen to remove the excess OsBp. Red, solution stayed at −5 °C for 20 days. Green, the same solution (red trace) left for 5.5 additional hours at 25 °C and analyzed to show more degradation. The degradation peak exhibits the same mt as A_10_U(OsBp)A_9_, as shown by spiking the corresponding oligo, but a comparable mt does not constitute identification proof.

In the first set of experiments, the reaction of A_10_CA_9_ with OsBp was monitored by CE using a long capillary. There is a clear decrease in the substrate peak and a clear increase in the product peak. The two isomeric products are not resolved in this case, so only one product peak was observed. Osmylation after 2 h in 13.6 mM OsBp yielded a CE profile shown in Figure 10 where a small broad peak, measuring 1% of the total, appears at a later mt. Stability studies for 20 days at −5 °C and for an additional 5.5 h at 25 °C yielded a steady increase of the peak area. The stability studies under the above conditions with A_10_C(Me)(OsBp)A_9_, A_10_T(OsBp)A_9_, and A_10_U(OsBp)A_9_ showed no new peak and no other detectable changes (see Supporting Information File 1, Figures S1, S2, and S3, respectively).

**Figure 11:**
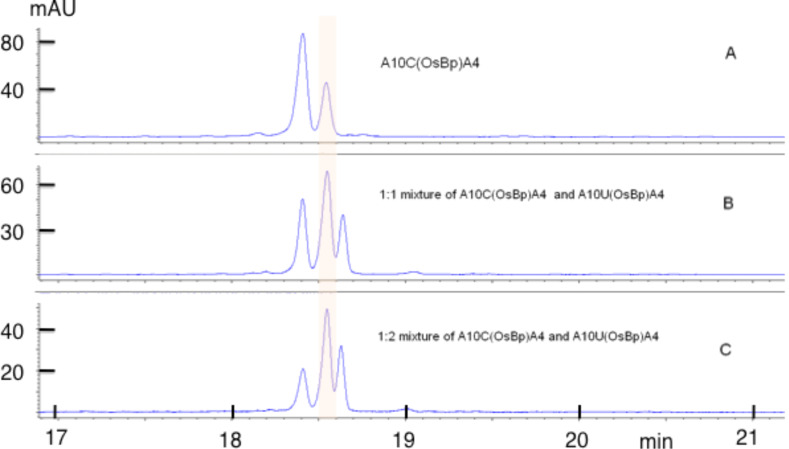
CE profiles: (a) the two peaks corresponding to the two topoisomers of A_10_C(OsBp)A_4_ (C-oligo) by top or bottom addition of OsBp to the pyrimidine ring; CE profile of A_10_U(OsBp)A_4_ (U-oligo) is comparable. (b) shows that a mixture of the two materials does not completely resolve to four peaks, but only to three peaks, even after using the long capillary (see Experimental). (c) shows that by adding extra U-oligo, the first peak decreases. Hence the U-oligo migrates later compared to the C-oligo. The profiles are shown in different scales to partially correct for the fact that by adding the U-oligo, the C-oligo is diluted. This figure illustrates that degradation of C(OsBp) to U(OsBp) in this oligo would have been detectable as a new peak appearing right after the smaller of the two isomers (see Figure 12 for further evidence).

**Figure 12:**
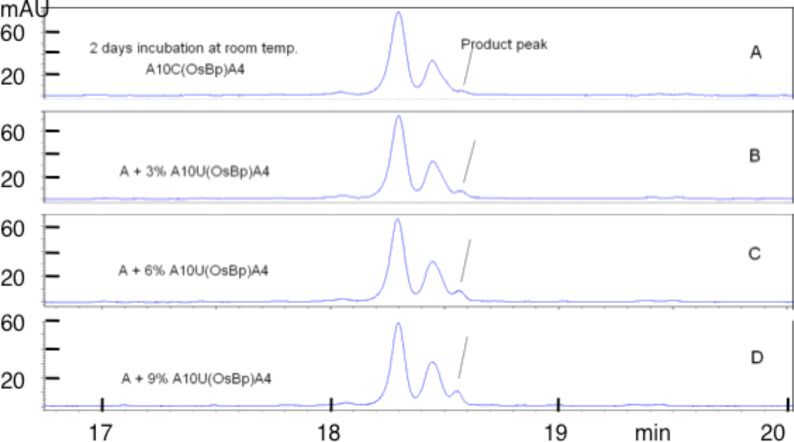
CE profiles: (a) after two days of incubation at room temperature a small shoulder appears at the mt position where the corresponding U-oligo would appear, suggesting negligible, if any, degradation of C(OsBp) to U(OsBp). (b–d) confirm by spiking with increasing amounts of U-oligo that this minor product/degradant peak comigrates with the smaller of the two isomers of A_10_U(OsBp)A_4_, and its increase over time would have been clearly detectable.

In an attempt to establish whether or not the observed degradation can be attributed to deamidation and hydrolysis of C(OsBp) to U(OsBp), we explored the stability of A_10_C(OsBp)A_4_ by CE using the same long capillary. The starting oligo was completely consumed in 13.6 mM OsBp and formed two product peaks migrating ahead of the intact oligo. A comparable result was obtained by osmylation of A_10_UA_4_. The two product peaks are attributed to the two isomers that are now resolved by CE because of the shorter length, 15-mer vs 20-mer. Still CE partially resolves the two isomers of A_10_C(OsBp)A_4_ from the two isomers of A_10_U(OsBp)A_4_; upon mixing, three peaks were observed instead of the expected four (see Figure 11). Over a period of three days at room temperature degradation was observed that measured less than 3% (see Figure 12).

**Figure 13:**
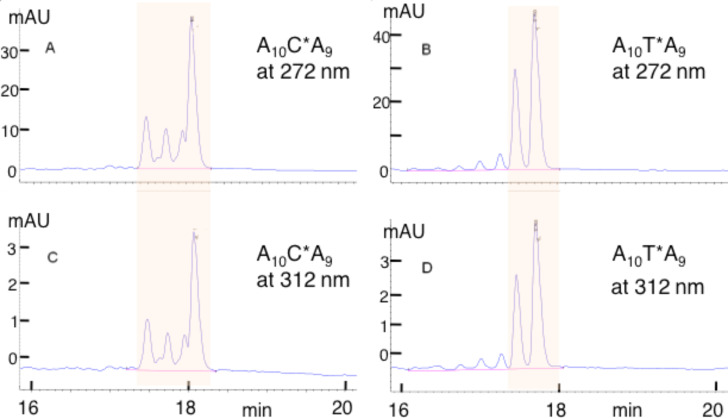
HPLC profiles of weeks-old-aged osmylated oligos. C* is an abbreviation for C(OsBp) and T* is an abbreviation for T(OsBp). A_10_T(OsBp)A_9_ was stable over weeks at room temperature, in contrast to A_10_C(OsBp)A_9_ that over time shifts materials to a new, unidentified, product (see major peak with the longest rt in (a) and (c)). Shaded areas correspond to degradation products of the C-oligo and stable osmylation products of the T-oligo. Smaller peaks outside the shaded region correspond to osmylation products of the impurities. The figure illustrates that all the products formed by aging of C(OsBp) carry the OsBp moiety. This is evidenced by comparing the values of *R* = 0.11 for the C-oligo (peak area in (a) divided by the peak area in (c)) with the value *R* = 0.11 for the T-oligo (peak area in (b) divided by the peak area in (d)); *R* = 0.01 for DNA that is an order of magnitude lower compared to the reported values here.

The above reaction (A_10_CA_4_ + OsBp) was reran using 5.5 mM OsBp (in order to slow down the kinetics) and monitored by IE HPLC. The disappearance of the intact oligo and the simultaneous appearance of two peaks eluting earlier was observed. These two peaks are attributed to the expected A_10_C(OsBp)A_4_ isomers (see Supporting Information File 1, Figure S4). In addition to the expected products, a small peak eluting after the starting material continued to increase with time. After 2 h, this peak measured about 2% of the total material. It should be noted that IE HPLC did not resolve A_10_C(OsBp)A_4_ from A_10_U(OsBp)A_4_. Hence, the observed, minor side product is not deamidation. The observed late peak grows over time and becomes the major component in room temperature stability samples. Similar observations were made with A_10_C(OsBp)A_9_, but not with any of the other oligos tested; hence only C(OsBp) degrades to a product not likely to be U(OsBp). A several-weeks-aged solution of A_10_C(OsBp)A_9_ is shown in the HPLC profiles of Figure 13 and illustrates the presence of a major decomposition product. Based on the literature, this degradation product may have been trans-uridine glycol [37], but that transformation requires that the OsBp moiety is labile. Figure 13 shows the profiles obtained at 272 nm and 312 nm and clearly indicates that each peak/product has the expected *R* = 0.11, strongly suggesting that OsBp is not lost upon standing. Comparable profiles are shown in Figure 13 for aged solutions of A_10_T(OsBp)A_9_ with no detectable changes. The stability of A_10_U(OsBp)A_4_ was also tested over a period of days and showed no detectable changes, suggesting that a transformation of C(OsBp) via U(OsBp) to the observed major degradant is not likely. It should be noted that all of the above stability tests were done in pure water, as the osmylation reaction is carried out in pure water. Further investigation into the stability of C(OsBp) containing oligos as well as the stability of C(Me)(OsBp) oligos should be done, and major degradants should be identified by NMR and mass spectrometry, but the corresponding reactions need to be carried out in buffers, because most of this instability is known to be pH-dependent [37]. For the purposes of sequencing osmylated DNA via nanopores, one may conjecture that tentative degradation of osmylated C under current labeling conditions of 2 h incubation time in 12–15 mM OsBp at room temperature yields 1–2% of alternative material that still carries the OsBp group. How this affects the quality of base calling still remains to be determined.

Even though there is no precedent in the literature that OsBp could react in a bifunctional way and yield inter- or intra-strand dimerization and/or polymerization of the reacting nucleic acid, this was investigated possibility as follows: pGEX3’-A_9_ was employed, which is a 32-mer long deoxyoligo, and both aged and fresh OsBp stock solutions were used for labeling. The reactions were monitored by IE HPLC and the method was adjusted to show the appearance of tentative 64 nucleotide long byproducts. No such long oligos were detected, even when the reaction was conducted with the highest concentration of OsBp for three times longer incubation compared to protocol B.

### Estimate for unreacted pyrimidines (false negatives)

To ensure reproducibility, a reaction must be exploited under pseudo-first-order conditions. Under such conditions, i.e., 20–25-fold excess of OsBp over the reacting nucleobase in DNA, reactivity depends on OsBp concentration, but it is independent of the actual concentration of the reacting nucleobase (N). Hence, the fraction of unreacted N (pyrimidine in this case) can be calculated using the first-order rate equation (Equation 1) from the observed rate of reaction, *k*, for a specific OsBp concentration, and the incubation time, *t*.

[1]
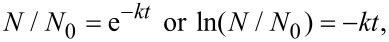


where *N* is unreacted, *N*_0_ is the initial concentration, and *t* is the incubation time. The half-life of a reaction (*t*_1/2_) is by definition when *N*/*N*_0_ = 0.5, or ln(0.5) = 0.693, with *t*_1/2_ = 0.693/*k*, or *k* = 0.693/*t*_1/2_. Therefore, Equation 1 transforms to Equation 2 as:

[2]
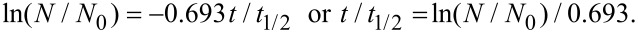


Equation 2 provides an easy way to calculate multiples of half-lives necessary to reach a certain level of reactivity. For example, for N/No = 1/1000 incubation time t = 10 half-lives, for N/No = 10,000, t = 13.3 half-lives and for *N*/*N*_0_ = 1/100,000, *t* = 16.6 half-lives, etc.

Equation 2 applies to any first-order or pseudo-first-order reaction. Hence the incubation time *t* necessary to reach a fraction of unreacted T or C equal to 1/10,000 is 13.3 half-lives of rate for T or C, respectively. We have repeatedly found that T osmylates with a rate of about 0.045 per min in the presence of 3.2 mM OsBp (protocol A) and C osmylates with a rate of about 0.08 per min with 12.6 mM OsBp (protocol B). These rates yield half-lives of about 15 min for T and 9 min for C at the corresponding OsBp concentrations. Hence 13.3 half-lives for C osmylation corresponds to 120 min and this is the incubation time recommended for protocol B. Evidently, after employing protocol B, intact T is orders of magnitude less than 1/10,000, because of its higher reactivity compared to C. On the contrary, protocol A aims to yield the minimum amount of osmylated C, while osmylating over 90% T. This is why protocol A exploits a 60 min incubation time with 3.2 mM OsBp, which yields from Equation 1: ln(*N*/*N*_0_) = −2.7 and *N*/*N*_0_ = 0.067, i.e., 6.7% intact T, or 93.3% osmylated T. It is advisable that new stock solutions of OsBp, as well as aged OsBp solutions, are evaluated for consistent reactivity.

## Conclusion

The experiments reported in this study strongly support the proposal that OsBp is, from a chemistry point-of-view, an outstanding agent to label pyrimidines in deoxyoligonucleotides. Whether or not OsBp is equally fit for translocation experiments that yield sequencing information remains to be shown. Our experimental set-up allows the determination of upper levels of purine osmylation and DNA backbone degradation under the proposed conditions. Approximate upper limits of 1/100,000, 1/100,000, and 1/1,000,000 were obtained for G-osmylation, A-osmylation, and DNA backbone cleavage, respectively. These upper limits were obtained using oligodeoxynucleotides and are likely to be valid with oligoribonucleotides and long nucleic acids because the osmylation reaction appears to be confined to the actual pyrimidine ring and independent of strand length and composition. Under the current protocol B, for practically 100% osmylation of T + C, levels of unreacted C are estimated at 1/10,000 and levels of unreacted T are negligible. Whereas osmylated deoxy T, (Me)C, and U are stable at room temperature over weeks, osmylated C exhibits degradation of 1–2% during the labeling protocol B. Osmylated C continues to degrade in water over time to products all carrying the OsBp moiety; the major degradant being inconsistent with C to U deamidation/hydrolysis. Due to C(OsBp) degradation, osmylated DNA needs to be stored at −20 °C. In summary, the results presented here provide improved protocols for the preparation of osmylated DNA, an overview of the upper limits in false positives and false negatives, and define the limits for accurate representation of the parent DNA in sequencing efforts.

## Experimental

### Materials

HPCE grade solution of 50 mM sodium tetraborate (pH 9.3) was purchased from Agilent Technologies. A 4% aqueous osmium tetroxide solution in 2 mL ampules was purchased from Electron Microscopy Sciences. 2,2’-bipyridine, 99+% (bipyridine) was purchased from Acros Organics. Oligos were ordered from Integrated DNA Technologies (IDT), some desalted and others PAGE purified. Circular ssDNA M13mp18 was purchased from Bayou Labs [35]. Oligos and DNA were diluted with DNase/RNase-free water (from MP Biomedical) to desired μg/μL concentration or to 100 μM stock solutions. Oligo solutions were stored at −20 °C. The purity of purchased oligos was tested by CE analysis and was typically better than 85%. The oligos used in this study, abbreviations, values of *R* of the corresponding osmylated derivative, and sequences are listed in Table 1.

### Preparation of OsBp stock solutions and protocols for osmylation reactions

Typically, 1:1 mixtures of OsO_4_ and 2,2’-bipyridine were prepared. A stock solution of OsBp at 15.75 mM OsO_4_ was prepared by mixing a 2 mL 4% OsO_4_ (ampule) with 18 mL of water that contained the equivalent 15.75 mM of 2,2’-bipyridine. The solubility tests in water were performed visually using weighted amounts of 2,2’-bipyridine, and its solubility was estimated at about 21 mM at room temperature. The solubility of 2,2’-bipyridine was also tested in solutions with increasing concentration of OsO_4_, and did not measurably increase over 21 mM, confirming that OsBp does not form a very stable complex. Typically, stock solutions of OsBp were prepared as 1:1 mixtures, and the use of saturated 2,2’-bipyridine solutions was abandoned [34] because of the difficulty in assuring that all the suspended 2,2’-bipyridine was removed. Due to OsBp reactivity, paper filters should not be used to filter OsBp, and plastic vials should also not be used to centrifuge OsBp. The stock solutions were dispensed in small, clear glass vials, sealed with parafilm, and kept at −20 °C until use; no detectable change in reactivity was observed with solutions that were stored sealed at 4 °C for four weeks. The experiments were initiated by mixing the OsBp stock solution and the oligo stock solution directly in a CE glass vial or HPLC vial or another stoppered glass vial filled with the appropriate amount of distilled water. The final concentration of [OsO_4_] = [2,2’-bipyridine], reported as [OsBp] is listed with each experiment. No buffer was added and reaction mixtures were incubated at 25 ± 2 °C. Analyses were carried out automatically. The aliquoting time together with the product distribution, in the form of an electropherogram or chromatogram, were recorded by the CE or HPLC. Spin columns (TC-100 FC, TrimGen) were used to remove excess OsBp according to the manufacturer’s instructions. Practically 100% recovery of labeled oligo and removal of up to 15 mM OsBp down to detectability levels after one or two purifications was achieved. Protocol A refers to 60 min incubation with 3.2–3.4 mM OsBp at room temperature and protocol B refers to 120 min incubation with 12.6–15 mM OsBp at room temperature. An earlier version of protocol B exploited 12 h labeling at room temperature, but recent results have shown it to be unnecessarily long. Protocol A results in over 90% T osmylation and less than 8% C-osmylation and protocol B results in 100% T + C osmylation.

### Capillary electrophoresis methods and analyses

Analyses of the reaction mixtures were carried out using an Agilent 1600 CE instrument equipped with a diode array detector (DAD) and Chemstation software, Rev.B.04.02 SP1, for data acquisition and processing. Only glass-type CE vials were used, as OsBp was found to react with polyurethane vials, lowering its effective concentration. Untreated fused silica capillaries (short: 50 μm × 40 cm and long: 50 μm × 72 cm) with an extended light path were purchased from Agilent Technologies.

The analysis of reaction mixtures was conducted using capillary zone electrophoresis (CZE) methods in 50 mM sodium tetraborate (pH 9.3) with 20 kV for the short capillary and 30 kV for the long capillary. The capillary temperature was typically 25 °C. The method associated with the short capillary was 18 min long and included a 3 min automatic buffer conditioning before sample analysis. The method associated with the long capillary was about 30 min long and included a 5 min wash. OsBp migrates early together with any other neutral compound present, the triphosphates of the nucleotides migrate last, and oligos/DNA in between. OsBp-labeled products migrate between the OsBp peak and the corresponding DNA peak. The resolution between the starting material and osmylated product, is among other parameters, a function of the oligo length and the number of reacting bases. With the longer DNA, a shift of the peak to earlier migration is detected as a function of the reaction progress. In CE profiles, the more abundant topoisomer migrates first ([30] and Figure 11), whereas in HPLC profiles, the most abundant topoisomer elutes last (Figure 6).

### High performance liquid chromatography methods and analyses

As a second analytical tool, we used an Agilent 1100/1200 LC HPLC equipped with a binary pump, DAD and a 1290 Infinity Autosampler/Thermostat, and Chemstation software Rev.B.04.01 SP1 for data acquisition and processing. Both the autosampler and the column compartment have individual temperature control. The method most suitable for oligo analysis is ion exchange (IE) with a salt gradient at 30 °C in the column compartment. A Dionex HPLC column, DNAPac 200PA, with a 2.0 μm inner diameter and 25 cm path length was used for the resolution of oligos based on both length and composition. Typically, a flow of 0.5 mL/min was used and a linear gradient system with solvent A of 25 mM Tris HCl at pH 7.0 and solvent B of 1.0 M NaCl in solvent A; the initial elution conditions were typically at 75% for A and 25% for B. The actual gradient slope, at about 1–1.5% B per minute, varied depending on the length of the oligos that needed to be resolved. Both CE and HPLC peaks were detected and identified using the DAD in the UV–vis region 200–450 nm and the electropherograms and chromatograms were recorded at several wavelengths, including 260 nm, 272 nm and 312 nm.

## Supporting Information

File 1 Additional figures are included to illustrate the stability of oligos with C(Me)(OsBp), T(OsBp) and U(OsBp) and the osmylation reaction of a 15-mer oligo monitored by IE HPLC.
