# Supplementary material for: False positives and false negatives measure less than 0.001% in labeling ssDNA with osmium tetroxide 2,2’-bipyridine
Source: Beilstein J Nanotechnol. 2016 Oct 12;7:1434–46. doi: 10.3762/bjnano.7.135 (PMC5082614; doi:10.3762/bjnano.7.135)
Supplement: File 1 — Additional figures are included to illustrate the stability of oligos with C(Me)(OsBp), T(OsBp) and U(OsBp) and the osmylation reaction of a 15-mer oligo monitored by IE HPLC. [file Beilstein_J_Nanotechnol-07-1434-s001.pdf]

Supporting Information

for

# False positives and false negatives measure less than 0.001% in labeling ssDNA with osmium tetroxide 2,2'-bipyridine

Anastassia Kanavarioti

Address: Yenos Analytical LLC, El Dorado Hills, CA, USA

Corresponding author

Email: Anastassia Kanavarioti - tessi.kanavarioti@gmail.com

**Additional Figures.**

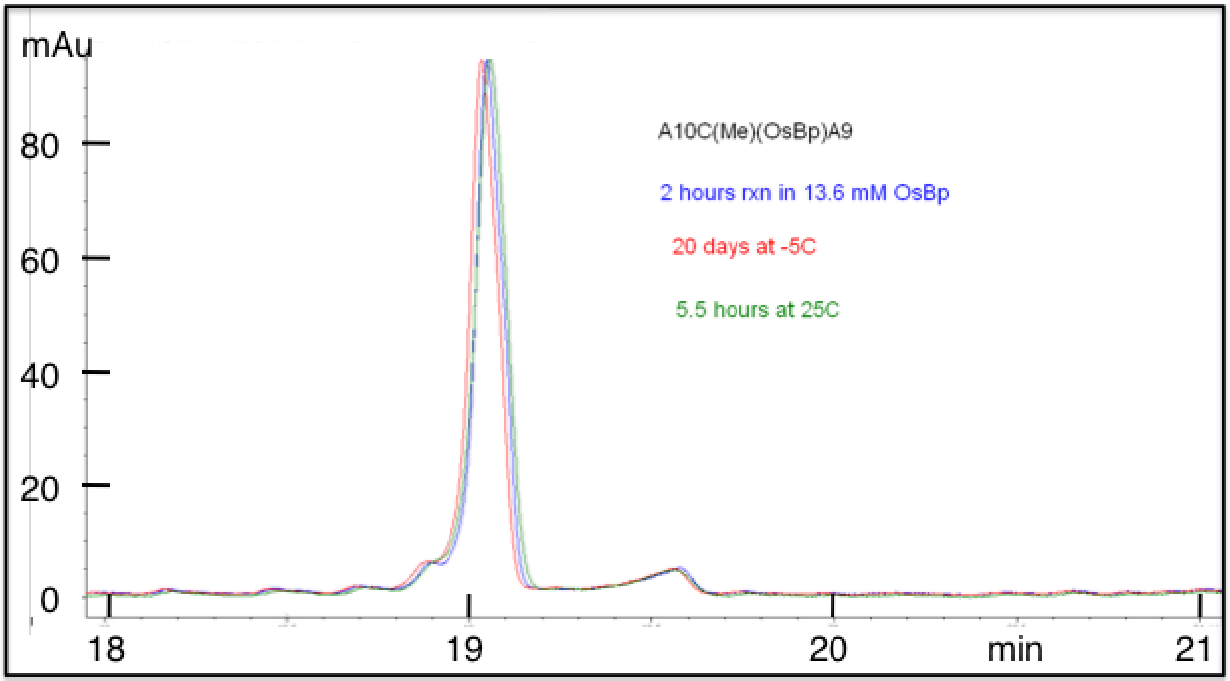


**Figure S1:** Excellent stability profile of C(Me)(OsBp) within the 20-mer oligodeoxyadenylate shown by CE analysis using the long capillary.

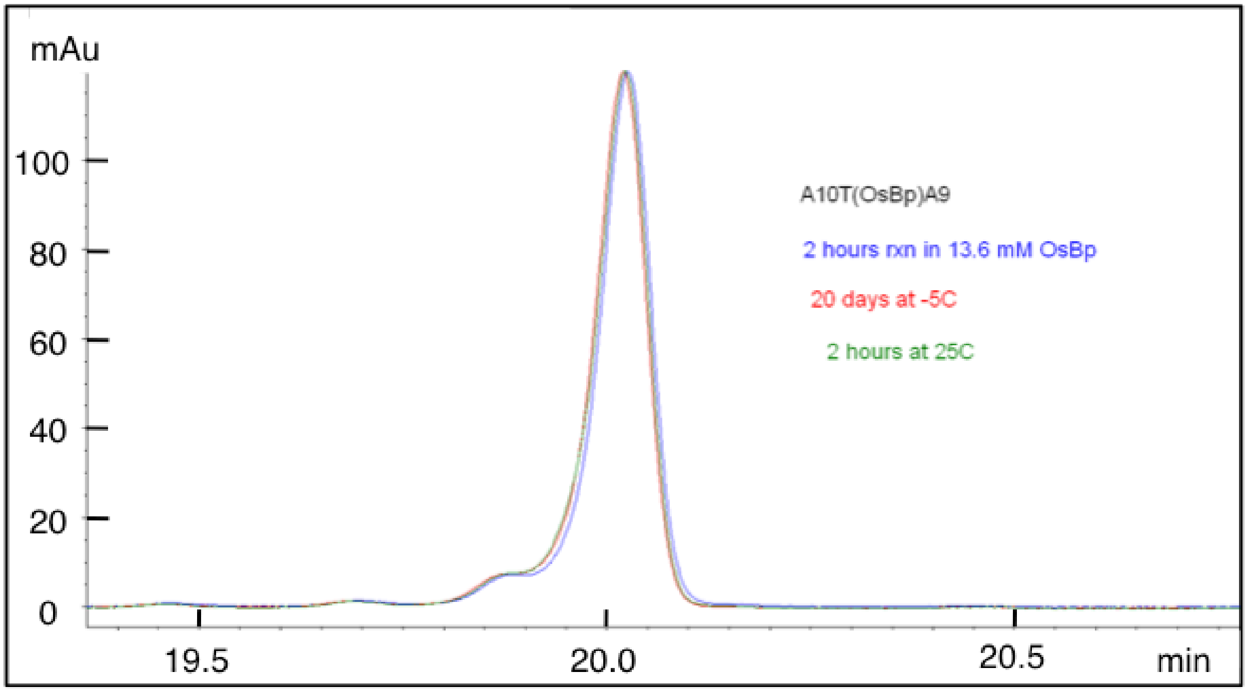


**Figure S2:** Excellent stability profile of T(OsBp) within the 20-mer oligodeoxyadenylate shown by CE analysis using the long capillary.

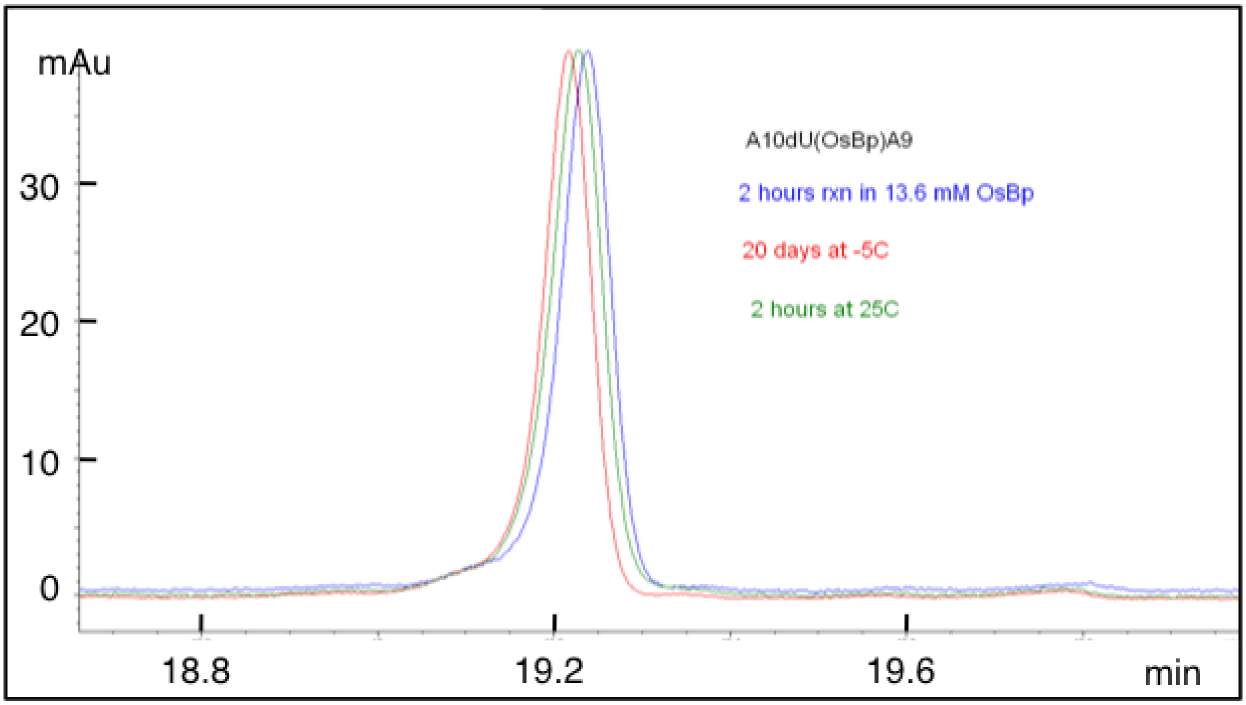


**Figure S3:** Excellent stability profile of dU(OsBp) within the 20-mer oligodeoxyadenylate shown by CE analysis using the long capillary.

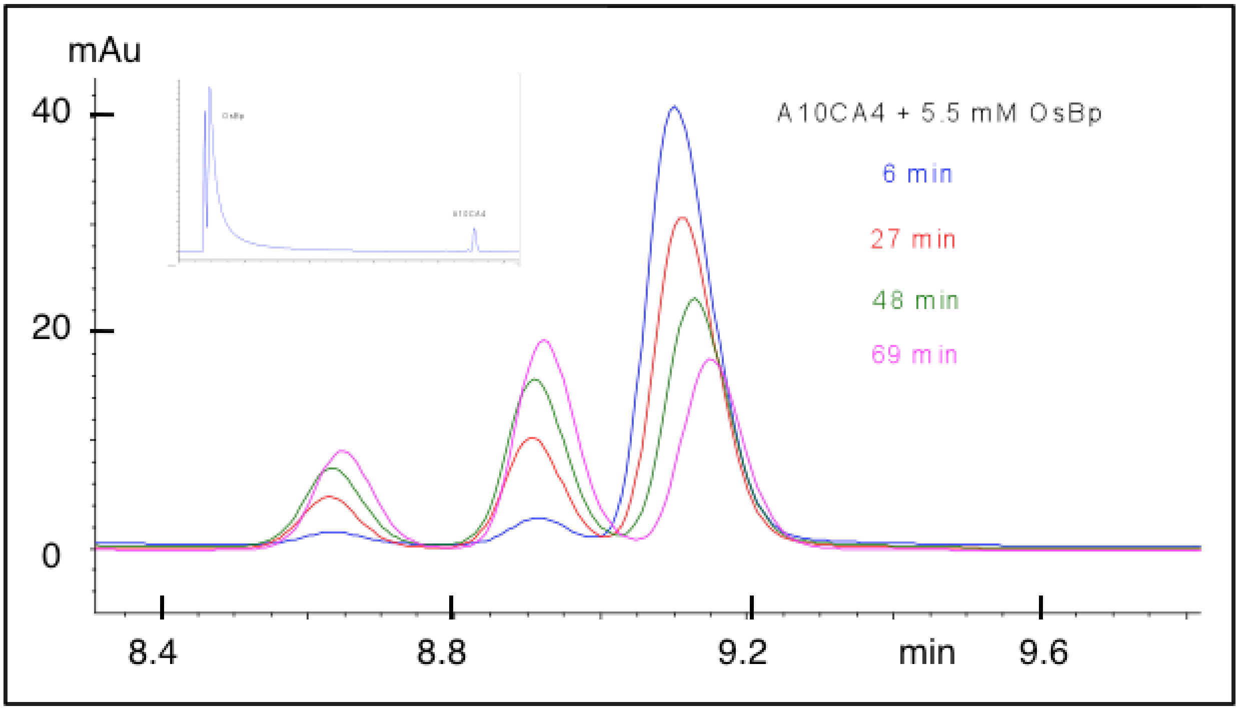


**Figure S4:** Reaction of 10 μM $A_{10}CA_4$ with 5.5 mM OsBp as monitored by IE HPLC analysis at 260 nm at about every 20 min. Reaction was conducted in 5.5 mM OsBp in order to slow it down, compared to the reaction in 13.6 mM OsBp. Slowing down the reaction does not change product distribution but allows one to observe the decrease of the oligo peak and the formation of two new peaks in about 2:1 ratio; these two peaks are the two topoisomeric osmylated products. The oligo does not absorb at 312 nm, but the two products do absorb (not shown here). The insert represents the analysis at 6 min and includes the full chromatogram in order to show the OsBp peak that is substantially larger compared to the oligo peak, but does not interfere with it. With time degradation peaks appear (not shown here) in the range 9.2 to 9.4 min comparable to the ones identified in Figure 13 from the osmylation of $A_{10}CA_9$.
